# I Don't Believe It, But I'd Better Do Something About It: Patient Experiences of Online Heart Age Risk Calculators

**DOI:** 10.2196/jmir.3190

**Published:** 2014-05-05

**Authors:** Carissa Bonner, Jesse Jansen, Ben R Newell, Les Irwig, Paul Glasziou, Jenny Doust, Haryana Dhillon, Kirsten McCaffery

**Affiliations:** ^1^Screening and Test Evaluation Program (STEP)Sydney School of Public HealthThe University of SydneySydneyAustralia; ^2^Centre for Medical Psychology and Evidence-Based Decision-Making (CeMPED)The University of SydneySydneyAustralia; ^3^School of PsychologyUniversity of New South WalesSydneyAustralia; ^4^Faculty of Health Sciences and MedicineBond UniversityRobinaAustralia

**Keywords:** cardiovascular disease, prevention, risk calculator, risk assessment, risk perception, lifestyle, behavior change

## Abstract

**Background:**

Health risk calculators are widely available on the Internet, including cardiovascular disease (CVD) risk calculators that estimate the probability of a heart attack, stroke, or death over a 5- or 10-year period. Some calculators convert this probability to “heart age”, where a heart age older than current age indicates modifiable risk factors. These calculators may impact patient decision making about CVD risk management with or without clinician involvement, but little is known about how patients use them. Previous studies have not investigated patient understanding of heart age compared to 5-year percentage risk, or the best way to present heart age.

**Objective:**

This study aimed to investigate patient experiences and understanding of online heart age calculators that use different verbal, numerical, and graphical formats, based on 5- and 10-year Framingham risk equations used in clinical practice guidelines around the world.

**Methods:**

General practitioners in New South Wales, Australia, recruited 26 patients with CVD/lifestyle risk factors who were not taking cholesterol or blood pressure-lowering medication in 2012. Participants were asked to “think aloud” while using two heart age calculators in random order, with semi-structured interviews before and after. Transcribed audio recordings were coded and a framework analysis method was used.

**Results:**

Risk factor questions were often misinterpreted, reducing the accuracy of the calculators. Participants perceived older heart age as confronting and younger heart age as positive but unrealistic. Unexpected or contradictory results (eg, low percentage risk but older heart age) led participants to question the credibility of the calculators. Reasons to discredit the results included the absence of relevant lifestyle questions and impact of corporate sponsorship. However, the calculators prompted participants to consider lifestyle changes irrespective of whether they received younger, same, or older heart age results.

**Conclusions:**

Online heart age calculators can be misunderstood and disregarded if they produce unexpected or contradictory results, but they may still motivate lifestyle changes. Future research should investigate both the benefits and harms of communicating risk in this way, and how to increase the reliability and credibility of online health risk calculators.

## Introduction

Health risk calculators are widely available on the Internet, with outcomes ranging from overall mortality to specific diseases such as cancer, diabetes, and cardiovascular disease (CVD) [1]. In the context of CVD, risk calculators use a mix of clinical and lifestyle risk factors to estimate the probability of a heart attack, stroke, or death over a specific period of time [2]. This may be communicated as a percentage or the alternative risk format of “heart age”, where heart age older than current age indicates modifiable risk factors [3,4]. These calculators may impact patient decision making about CVD risk management with or without clinician involvement, but little is known about how patients use and understand such risk calculators. Previous research on diabetes and cancer risk calculators suggests that people may disregard results that do not match their prior risk perception [1,5], and the presented numerical format may affect perceived credibility of the results [6].

Clinical guidelines around the world advocate CVD risk assessment based on “absolute risk”—the percentage risk of a cardiovascular event over a 5- or 10-year timeframe [7]. The Framingham model is commonly used and accounts for the effect of non-modifiable risk factors, including age and gender, as well as modifiable risk factors, such as smoking, blood pressure, and cholesterol [8,9]. However, research has established that percentages are poorly understood by both clinicians and patients [10,11]. Clinicians also report situations in which communicating absolute risk to patients is unhelpful [12,13]. In particular, patients with lifestyle risk factors (eg, smoking or obesity) can have low percentage risk (eg, younger patients and women are likely to have low 5-year absolute risk<10%), which may reduce motivation to change lifestyle before it leads to CVD and other chronic illnesses [12]. Such communication issues may discourage GPs from using absolute risk assessment, contributing to the suboptimal use of absolute risk guidelines around the world [14,15].

Preliminary research suggests that converting percentage risk into an individual’s heart age may be a useful alternative for communicating CVD risk. A focus group study using hypothetical risk found that patients preferred heart age over other CVD risk formats, but there were concerns it may frighten people if older than their current age [16]. A randomized controlled trial found that giving patients a CVD risk profile, including heart age, improved cholesterol levels compared to usual care over the first year of cholesterol medication treatment, especially for higher risk patients [17]. The similar concept of “lung age” was found to motivate smokers to quit regardless of the result: normal lung age acted as an incentive to stop smoking and abnormal lung age sent a message that quitting could slow deterioration [18]. However, heart age and lung age were not specifically compared to percentage risk in these studies [17,18]. An experimental study found that heart age improved understanding of risk compared to 10-year percentage risk and had more emotional impact for younger people at higher risk [19]. A study on a New Zealand heart age tool suggests it may increase clinician understanding and confidence in assessing absolute CVD risk, but patient outcomes were not assessed [3].

To the authors’ knowledge, there have been no studies investigating patient understanding of heart age compared to 5-year percentage risk, which is currently used in Australian guidelines and online tools. Nor have there been any studies investigating the best way to present heart age. This study aimed to investigate patient experiences and understanding of online heart age calculators that use different verbal, numerical, and graphical formats, based on 5- and 10-year Framingham risk equations [8,9], which are used in clinical practice guidelines in many countries around the world [3,7,20,21].

## Methods

### Ethical Approval

Ethical approval for the study was obtained through the Human Research Ethics Committee of the Sydney Local Health District (Protocol No. X11-0200). Each participant gave written consent before participating in the interview.

### Recruitment

**Figure 1:**
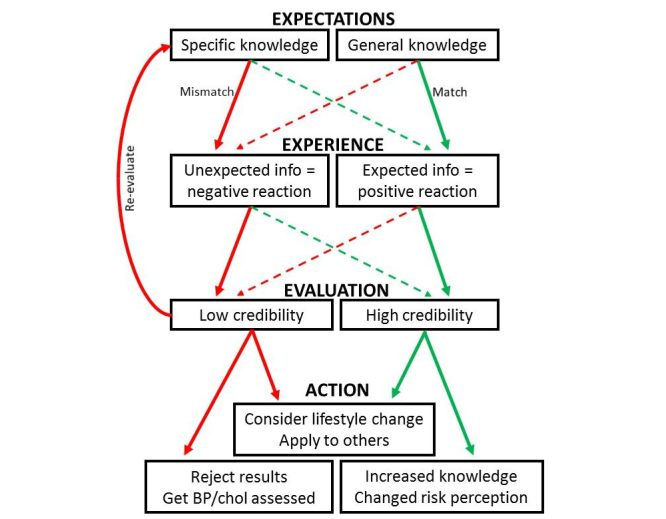
Process of using risk calculators: red arrows indicate low credibility pathways, green arrows indicate high credibility pathways, solid lines indicate main pathways identified, dashed lines indicate alternative pathways identified.

General practitioners (GPs) in New South Wales, Australia, recruited 26 patients between 40-70 years of age, with at least one CVD or lifestyle risk factor, who were not currently taking medication, targeting low (5-year absolute risk<10%) to moderate (10-15%) risk patients who may be less motivated by their percentage risk result [20,21]. Purposive sampling was used to recruit participants with a range of ages, gender, knowledge of risk factors, and risk calculator results (see Table 1). This was done by modifying the eligibility criteria given to recruiting GPs throughout the study. Analyses based on 26 participants suggested saturation of key themes (see Figure 1), so no further recruitment was conducted [22].

**Table 1:** Participant characteristics in order of absolute risk result, by gender.

ID	AR^a^	Gender	Age	HA^b^: Unilever^c^	HA: New Zealand^d^	HA vs age	Knew SBP^e^	Knew Chol^f^
102	1%	Woman	48	46	<48	younger	Y	Y
109	1%	Woman	52	46	<52	younger	Y	N
112	1%	Woman	54	49	<54	younger	Y	N
99	2%	Woman	40	51	64	older	N	N
118	2%	Woman	51	51	61	mixed	N	N
68	3%	Woman	57	43	59	mixed	Y	Y
87	4%	Woman	57	61	64	older	Y	Y
115	4%	Woman	63	62	69	mixed	Y	Y
108	4%	Woman	67	62	68	mixed	Y	N
103	6%	Woman	39	39	39	same	N	N
71	6%	Woman	57	53	<57	younger	Y	Y
70	6%	Woman	58	74	72	older	Y	Y
107	8%	Woman	49	59	60	older	Y	Y
119	8%	Woman	59	79	70	older	N	N
116	9%	Woman	60	80	73	older	N	Y
106	10%	Woman	66	72	>75	older	N	N
84	3%	Man	45	51	52	older	Y	N
91	3%	Man	48	52	58	older	N	N
111	4%	Man	50	60	57	older	Y	N
63	5%	Man	55	57	66	older	N	N
96	5%	Man	58	63	62	older	Y	Y
113	6%	Man	41	43	46	older	Y	N
110	7%	Man	62	58	63	mixed	Y	Y
94	8%	Man	60	74	69	older	Y	N
95	11%	Man	58	60	70	older	N	N
65	12%	Man	55	60	66	older	N	Y

^a^AR: initial 5-year absolute risk estimate on New Zealand calculator (<10% indicates low risk; 10-15% indicates moderate risk)

^b^HA: heart age result on each website

^c^Unilever: website developed by Unilever [23]

^d^New Zealand: website developed by New Zealand Heart Foundation [24]

^e^SBP: systolic blood pressure, Y=yes, N=no

^f^Chol: cholesterol, Y=yes, N=no

### Participants

Participants were 16 women and 10 men, between 40 and 67 years of age, with a range of highest educational attainment: 4 had not completed high school, 6 had completed high school, 7 had a technical qualification, and 9 had completed a university degree. Five-year absolute risk results ranged from 1-12%, with 23 at low risk (<10%) and 3 at moderate risk (10-15%) of a CVD event. Compared to current age, the heart age results were: 16 older, 4 younger, 1 same as current age, and 5 mixed results for the two calculators.

### Process

**Figure 2:**
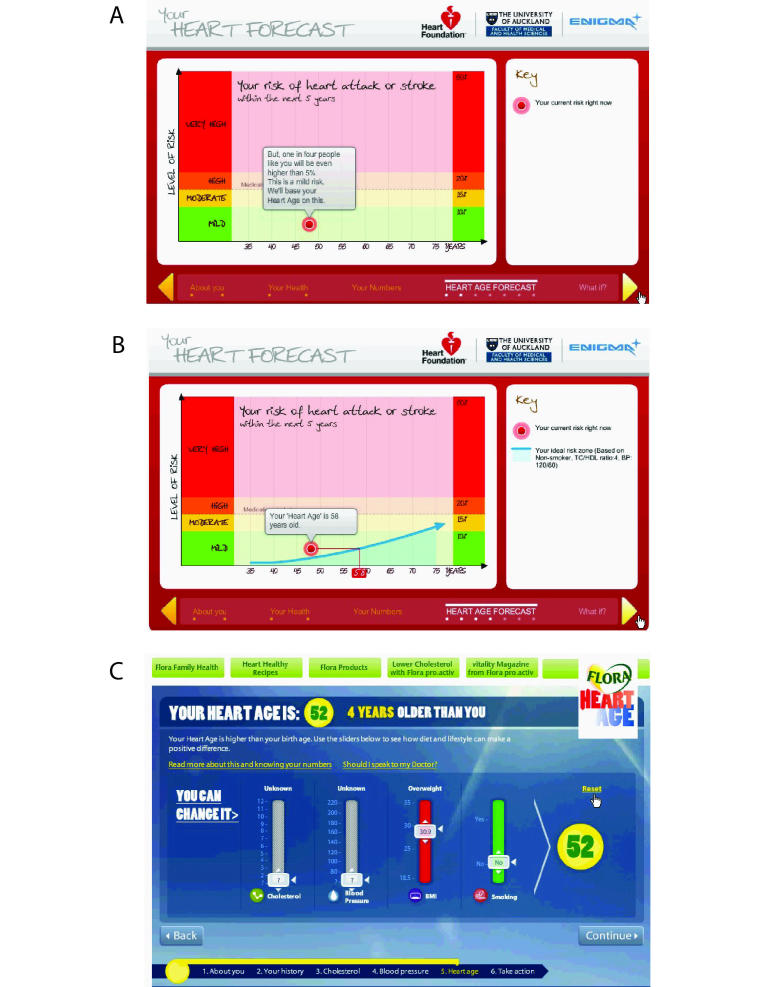
Example of heart age calculator results for ID91: male, age 48, BP and cholesterol unknown. A: New Zealand, initial absolute risk result 3% but estimate increased to 5%; B: heart age 58; C: Unilever result, heart age 52.

Two heart age calculators based on Framingham risk equations were publicly available at the time of the study in 2012 (see Table 2 and Figure 2) [23,24]. A protocol including think aloud and semi-structured interview methods was developed based on past research showing that a concurrent think aloud protocol elicits more information, but additional insights can be gained retrospectively (see Multimedia Appendix 1 for protocol) [25]. The interviewer (CB) was a researcher trained in public health qualitative methods, who piloted the protocol with a convenience sample of 4 participants who met the study eligibility criteria. Pilot participant feedback was discussed with the research team and the think aloud instructions were clarified before commencing the study. Participants were asked to think aloud as they used each website in random order, with minimal input from the interviewer unless they had difficulty using the website. No interpretation of the results was provided until the end of the interview and the interviewer clarified that the researchers were not connected to the websites if this issue arose. In order to practice thinking aloud consistently, participants completed a simple “spot the difference” task in which they described what they were doing. Upon successful completion of this practice task, participants began using the heart age calculators. A “keep talking” sign was placed above the computer, which the interviewer would point to if the participant was silent for more than 10 seconds. The entire session was audio-recorded and use of the websites was video-recorded using SMRecorder screen capture software [26]. All interviews were audio-recorded, but technical problems prevented the use of screen capture software in some interviews: 3 participants had no video data because of computer or software issues and 5 participants had only one calculator video-recorded (3 due to computer or software issues and 2 due to the Unilever website being taken down, who completed the study over the phone when the website became temporarily available again).

**Table 2:** Main differences between the two heart age calculators.

Variable	New Zealand Heart Foundation [24]	Unilever [23]
Timeframe	5-year risk based on cholesterol Framingham risk equation [9].	10-year risk based on cholesterol or body mass index Framingham risk equation [8].
Minimum heart age	“Lower than” current age. Current age is the lowest value shown.	Heart age result can be younger than current age.
Include % risk	Yes – % and risk level (mild, moderate, high, very high).	No – heart age only.
Graphical display	Yes – trajectory over age with colors indicating risk level.	No – text only.
Risk factors asked about	Age, gender, family history, smoking, diabetes, systolic blood pressure, cholesterol ratio, ethnicity.	Age, gender, family history, smoking, diabetes, systolic/diastolic blood pressure, total/HDL cholesterol, height, weight, waist.
Modifiable risk factors at final results page	Blood pressure, cholesterol, smoking, diabetes (if not already diagnosed).	Blood pressure, cholesterol, smoking, body mass index.
Missing data	If blood pressure and/or cholesterol values were not known, two values were given: a population average based on demographics, and a higher than average value that “1 in 4 people like you” would have. These estimates were used to calculate two absolute risk results (see Table 1 for initial result based on the average). The higher than average value was used to calculate heart age.	If blood pressure and/or cholesterol values were not known, alternative Framingham-based algorithms including body mass index were used to calculate heart age [8].

### Analysis

A framework analysis method was used to analyze the interview transcripts, which involved five steps [27]. The first step was familiarization with the data: CB read through all 26 transcripts, recorded the calculator input/output for each participant from videos if available or from transcripts and field notes if unavailable, and discussed this and 2 transcript excerpts with all authors, covering younger and older heart age results. The second step was to create a thematic framework: CB, JJ, and KM read a sample of 5 transcripts covering younger, same/mixed, and older heart age results, and developed the initial framework. Third was indexing: CB, JJ, and HD each watched a video to ensure understanding of the process and coded the remaining transcripts according to the framework, with new themes and revisions to the framework discussed (see Multimedia Appendix 2 for final framework). Ten transcripts were double-coded independently by two researchers. The fourth step in the analysis was charting: CB, JJ, and HD summarized themes and supporting quotes from each transcript in the framework (a matrix with participants as rows and themes as columns), with transcripts reread and discussed to resolve any disagreement about the best way to represent the data. The fifth step was mapping and interpretation: CB and JJ examined the framework within and across themes and participants to identify overarching themes and relationships, independently summarized the process of using and interpreting the two risk calculators in a diagram, and differences between the 2 process diagrams were discussed with KM and HD. The order in which the calculators were viewed was taken into account, based on separate coding of the two websites, but this did not appear to influence the overall process. Then JJ, HD, and KM each read 2 additional transcripts to check the findings and the final process diagram was created (see Figure 1). The final results were discussed with all authors. Rigor was addressed by: repeated coding of transcripts by different team members to ensure a comprehensive themes list and framework was achieved; an iterative process of constant comparison between the existing framework and new data; detailed documentation of the analysis process; and discussion of emerging and final themes with all authors [28].

## Results

### Reliability

The reliability of the risk calculator results was affected by several factors. Misinterpretation of risk factor questions was common, with 9/26 participants making at least one error in their responses to the questions, based on inconsistencies between the data entered in the two calculators and their thought process while entering the data. Many participants couldn’t remember their exact blood pressure or cholesterol levels but were aware that these risk factors were low or normal based on past assessment, and this did not match the estimates provided by the calculator.

I guess if I’d known my blood pressure and cholesterol level, it might have been lower but that's all right…automatically went to the default position, the higher setting… I think I’d be lower than that in reality.

ID95, older heart age

The two calculators gave different heart age results for all but one participant due to these input errors and the different assumptions built into the calculators: the use of different absolute risk models (5- vs 10-year risk; use of body mass index if cholesterol was unknown), the estimates used when blood pressure was unknown, and the “ideal” that participants were compared to when calculating the heart age.

### Risk Formats

In the New Zealand calculator, the explanation of the percentage risk information was often overlooked, with 12/26 participants skipping through at least one part of the explanation. Even when the percentage information was seen, it was often forgotten by the end, with participants generally more focused on the graph and heart age results. When specifically asked, many participants were unsure what the percentage referred to, even though the graph title clearly stated it was the percentage risk of a heart attack or stroke in the next 5 years.

Well, that I have a 2%, so I have 2. Well, what does it mean? Does it mean that I, 2 days out of 100, I’m at risk of a heart attack. I don't know what that means. I have a 2% chance, I have a…well, it sounds low but what does it mean? I mean I don't know…No, I think the heart age was good.

ID118, mixed heart age results

The graph in the New Zealand calculator was sometimes confusing, particularly the first few screens where many different numbers were used in the explanation of the results (see Figure 2A). However, other participants liked the graph and demonstrated a clear understanding of how risk would increase with age when viewing it.

Yeah, I think the graph is not so great…it is more the older generation I guess you’d be looking at as well, not people who are really computer savvy and they can read 100 things on the screen at one time and take it all in.

ID107, older heart age

The risk is going to go up anyway and even if I stay doing what I’m doing now, it’s still going to rise, and to keep it at the lowest risk I need to do more…being vigilant on the blood pressure and the cholesterol and just being probably a bit more active.

ID115, mixed heart age results

The heart age format in both calculators was much more salient, meaningful, and elicited more emotional reactions in participants. Younger heart age was seen as very positive and participants often preferred the calculator that gave them a lower heart age, although some felt it was unrealistic and could discourage people from improving their lifestyle. Older heart age was confronting and participants’ first reaction was often disbelief, particularly if they felt they had a good lifestyle. Heart age had a more immediate meaning to participants than percentage risk, indicating a healthy lifestyle if younger, and the need to change if older.

Oh, younger than me—that’s good news (laughter)…The fact that it’s younger. I mean I already feel that I am probably healthy-ish for my age, healthy. So I’m not assuming that yes, ok, my heart is 6 years younger than my body but I’m, to me that says, yeah, you’re ok.

ID109, younger heart age

Your heart age is 52? Get out of town…How does that work? Your current risk right now is there. But my heart age is 52…No cholesterol. Normotensive…Don’t believe it…not smoking, eating a healthy diet…I’m grumpy with this website already. Because it’s asking me to do things that it didn’t actually question me about before, like being active or eating.

ID84, older heart age

The multiple formats presented in the New Zealand calculator were sometimes perceived as contradictory, particularly low percentage risk in the green mild category compared to an older heart age result. The use of consistent colors was also important—green indicated a mild risk level in the basic graph, but a green line was also used to show how risk would increase if the participant started smoking or developed diabetes.

Well, off the basis of one number, you're saying I’m middle in the mild, yet you've automatically put me at 5 years older than my heart. So those two things are probably contradictory in a way…by clicking on the start smoking you strangely get a green line, which would indicate a good thing which is probably not right. Ah…it should be a black line.

ID113, older heart age

The ability to modify different risk factors to see what effect they had on the results also had mixed responses from participants: some weren’t interested in using it or didn’t understand it, while others spent some time playing around with the factors to reduce their heart age result or make the estimated risk factors more realistic. However, the message this conveyed depended on how the participant used it—for example, the following participant concluded that blood pressure was more important than cholesterol because she happened to move blood pressure to a higher level.

What about if you get diabetes and you smoke? You are dead by 55. Wow…Oh, so the cholesterol isn’t too bad. It’s, gauging from this, it’s when the blood pressure goes up and if you have diabetes. That’s what I’m getting from this.

ID103, same heart age as current age

Participants tended to focus on the risk factors that were most relevant to them; for example, the effect of smoking was of more interest to current/ex-smokers and several participants wanted to see the effect of alcohol.

Your projected risk if no changes are made…ok, so I go from a mild to a high if I don’t change anything that I do. All right…If I quit smoking long term…that decreases it…so I’m only in the moderate range then if I do that, ok…It's something that I have actually been thinking of for quite a while.

ID99, older heart age

What about drinking, where is the drinking? That would be more interesting to me…you would have to put drinking in there as we get older.

ID70, older heart age

### Process of Using Risk Calculators

The process of using the risk calculators involved several stages: expectations of CVD risk based on prior knowledge, experience of using the calculator, evaluation of the credibility of the results, and actions considered as a result of this process. This is illustrated in Figure 1, with examples in Tables 3 and 4. Participants’ expectations, experience, evaluation, and actions sometimes changed between the two calculators, but the order in which they were viewed did not appear to affect the overall process. The process diagram therefore describes the range of pathways that participants followed regardless of the order in which the calculators were used, with solid lines indicating the two main pathways, and dashed lines indicating alternative pathways.

The high credibility pathway tended to occur when participants had little prior knowledge of their own risk of CVD. Their general knowledge of CVD risk factors was consistent with the information in the calculators, and so they tended to accept the results and have a more positive reaction to them. Those who received a younger heart age result usually had a positive reaction. In these situations the credibility of the results was not closely questioned and led to various actions: participants considered changing their lifestyle to lower risk even further or maintain younger heart age, thought of higher risk family or friends who could benefit from using the calculator, and sometimes perceived increased understanding or changed risk perception.

Alternatively, those with more specific knowledge evaluated the calculator as having high credibility if they had a positive reaction to the calculator, such as getting lower heart age than current age or all the information matched what they knew about their CVD risk. Seeing a similar result for the second calculator also increased credibility.

The low credibility pathway tended to occur when participants had more specific knowledge of their own risk of CVD, but could not necessarily remember their exact blood pressure and cholesterol levels. These participants were more likely to encounter unexpected information in the calculators, and reacted negatively to receiving an unexpected heart age result. In these situations, the credibility of the results was questioned and participants re-evaluated their prior expectations (eg, that they already had a good diet) and experience of using the calculator (eg, that they weren’t asked any questions about diet) to make sense of the result. Common reasons used to discredit the results were the lack of relevant lifestyle questions in the New Zealand calculator and the influence of corporate sponsorship in the Unilever calculator. However, even when the results were rejected, participants still considered lifestyle change and felt that the calculators would be useful for others. Some decided they should get their blood pressure or cholesterol checked again to increase the accuracy of the risk assessment.

Alternatively, those with little prior knowledge evaluated the calculator as having low credibility if they had a very negative reaction to the results or specific components (eg, some men believed that body mass index was inaccurate for them). However, they still considered lifestyle change and getting a more accurate assessment.

In summary, using the heart age calculators appeared to lead participants to consider lifestyle changes regardless of the pathway they described and regardless of their heart age result. This is illustrated by the quotes in Tables 3 and 4.

**Table 3:** Examples of main pathways for low vs high credibility.

Main pathway for low credibility	Example: quotes from ID70 (woman aged 58, higher heart age, 5-year absolute risk 6%)	Main pathway for high credibility	Example: quotes from ID119 (woman aged 59, higher heart age, 5-year absolute risk 8%)
Expectations: Specific knowledge of own risk factors is less likely to match experience of using calculator	*Do you know your blood pressure – yes. Oh, well…I can't really remember but I’ll just put in I think it was 138 over 81…Do you know your cholesterol – can't remember. Oh, hang on a sec, 3 to 4…high, I think it was high…She said it was sort of middle – 5.*	Expectations: Having ^only^ general knowledge about CVD is more likely to match experience of using calculator	*I like to think that I am low (risk) but I don’t know. My mum did have some issues with her heart when, when she was young, I mean probably, oh, late 60s, early 70s. So, if it is something to do with genetics or whatever, well I’m getting into that age, so I don't know. I would say I’m in low. I would like to believe that.*
Experience: Negative emotional reaction when calculator doesn’t match expectations, leading participants to question credibility	*Current risk this is a mild – yeah…I agree with that...Your heart age is what, rubbish. I don't believe that…no way…What…I better start changing this, hadn’t I…Too in your face, I don't want to know that. I don’t want to know my heart age…72 years of age. Too frank… Terrible, 72...It sounds like I’m going to have a heart attack very soon...I’m on the way.*	Experience: If information makes sense and matches expectations, and/or elicits a positive reaction, credibility is not questioned	*You will be near this point, this is a mild risk, oh good (laughter)….I’m glad about that, happy, happy, happy…this risk will be your risk as you get older, ok. So I have to be careful what I do…Your ideal risk based on non-smoker, your heart age is 70 years old, oh wow (laughter)… You can reduce your risk of heart attack or a stroke by not smoking – I don't, eating a healthy diet – ok, by being active for at least 30 mins on most, on most days of the week – ok, I need to do that.*
Evaluation: Low credibility leads participants to re-evaluate expectations ^and^ experience	*Terrible, again it's terrible. I hate this 74 and 72, that’s not real…The only one that can say my heart age would be the, my cardiologist when he goes in and has a look at my heart age…A lot of things on the Internet really aren’t sort of factual...I reckon other people my age would probably be on a higher…two of my school chums would be definitely because they're overweight, they’re on the tablets.*	Evaluation: High credibility leads participants to consider several possible actions without re-evaluating expectations and experience	*Wow, this is very good…it’s an eye opener to, you know, I think that I didn't have, to be honest, I didn't have much problem with my heart…Oh yeah, I’m overweight and this and that but never thinking that it’s, it (would) have such an effect on my heart and that, yeah, I’m like anybody else. You know I can have a heart attack and I can have problems with my heart, scary…So the cholesterol level and the blood pressure is something that…I need to be very much aware and try to you know make sure that I check it all the time with the Doctor.*
Action: Lifestyle change and usefulness for others considered	*Yes, I think it is a wakeup call. Yes, I am watching my diet. Yes, I am not exercising enough yet but I will (laughter)… I think I might put Mum on here and give her a go.*	Action: Lifestyle change and usefulness for others considered	*My husband should do this…it’s telling me that I need to do something. That I have to take action…I don’t know if, if the heart can get back to, to match my age...that is something I need to talk to my Doctor about.*

**Table 4:** Examples of participants considering lifestyle change by heart age result.

Heart age result	Quote illustrating consideration of lifestyle change
Older heart age than current age	*Ok, so this is interesting...if I reduce my BMI...so weight is a clear factor here…This is quite good, this tool here, because it actually gives me some targets for my BMI and what sort of weight I should be. So it’s, man, I’m going to have to lose a lot of weight (laughter)...This is quite good because I think it, it clearly shows that my weight is something I need to work on. And I think the fact it’s red, it takes you straight to that and I do like that.* [ID65, older heart age]
Mixed results (younger then older heart age result)	*With that graph specifically, that it’s a general rise anyway without taking into account, you know it’s not going to be stable. It's automatically going up so you have to work a bit harder at it…that’s made an impression on me and that’s the biggest thing I’ve picked up that, yeah, you’ve still got to keep working at it. It doesn’t matter what you’re doing now that sort of just yeah maintain or get more, just to try and yeah reduce the risk, keep going to reduce it…just being aware of it and, and I think it, I’m pleased that it’s at the lower level but also you’ve got to be vigilant.* [ID115, mixed results]
Same heart age as current age	*When I went on the Heart Foundation one and I changed my cholesterol and it increased my risk of heart disease...that’s something that’s important to me, because it happens in my family, so, yeah. This sort of thing that I have sitting out here (biscuits) will not be happening. Well, it still will, but not to the extent that it does in this household...I would probably, you know, take out maybe one load of biscuits and put some carrots in.* [ID103, same heart age as current age]
Younger heart age than current age	*So if I move the cholesterol down to 4 and…that reduces me down to 48. So I think I better get myself cracking and...get my cholesterol down... I think it means I’m probably tracking ok, I’d prefer if it was lower so then you know that my cholesterol can get reduced. So I know how to do that it’s just that I haven’t done it...I like the idea of it being, I like the idea of it being 48 better than 53.* [ID71 , younger heart age]

## Discussion

### Findings and Implications

Our findings suggest that online heart age calculators prompt people to consider improving their lifestyle regardless of the accuracy and perceived credibility of the results, or the result they received. This is consistent with the findings of the “lung age” study, where both positive and negative results prompted smokers to consider quitting [18]. As found in previous studies on diabetes and cancer risk calculators, participants often disregarded unexpected or negative information [1,5], and actively sought reasons to discredit the results. However, participants in this study considered lifestyle change and felt the calculators would be useful for other people, even when they claimed to disbelieve the calculator result. The value of such tools is therefore dependent on their goal—if it is to prompt people to think about improving their lifestyle regardless of the level of risk [4], they appear to be achieving this; if it is to provide accurate information and understanding of risk, then they could be improved [1]. However, thinking about lifestyle changes does not necessarily translate into actual behavior change. Previous research has found that personalized risk calculators have limited impact on behavior [29], but they may prompt people to seek further information and support as an interim step before behavior change [30].

The ultimate goal of risk calculator websites will vary depending on the motivation and target audience of the organization that created it. Our findings show wide variation in the way that people use and understand features of personalized risk calculators, with implications for designers of such tools. Patients in our study misinterpreted risk factor questions, entered data inconsistently between different risk calculators, skipped information that may have prevented these issues, and did not use all features of the tools. Some of these issues may be improved by simple design alterations, such as larger font, easier navigation, and clearer instructions for how to use the risk factor modification tools. Alternatively, simpler, less interactive information formats may be more effective in terms of information processing and understanding of risk [31,32].

This study is consistent with previous findings that heart age elicits more emotional reactions and is more meaningful to patients than percentages [16,19]. All participants demonstrated good understanding of the effect of risk factors on heart age and its link to lifestyle, but the percentage information was interpreted in many different ways and often overlooked. This supports the large body of research showing that percentages are often misunderstood [10,11]. The downside of heart age was that it was very confronting for participants to receive a much older heart age than their current age and some explicitly said they would prefer not to know.

The presentation of multiple formats was problematic because low percentage risk and older heart age were perceived as contradictory, suggesting that the focus should be on explaining one or the other, not both. Although the New Zealand calculator was developed with a step-wise structure that attempts to fully explain how the results were calculated [33], the large amount of information with multiple numbers was confusing for many participants, and the simplified Unilever format was often preferred even though corporate sponsorship reduced its credibility. The graph used in the New Zealand calculator with color-coded risk categories and projected risk over age (see Figure 2) appeared to be useful additional information for many but not all participants. Since preferences for and understanding of different CVD risk formats were variable in our study, quantitative research is needed to test the effect of presenting heart age in different formats and identify the best way to present such information to different groups of people. Future research should include measurement of benefits like understanding and motivation to improve lifestyle, but also the potential harms of conveying such emotive information, including worry and seeking unnecessary tests for low risk.

Future research on online health risk calculators could also investigate how to increase their reliability and credibility. One option is to involve clinicians in explaining the results, which could improve perceived credibility and the accuracy of the risk factor data, and may prevent misunderstandings. However, since health consumers use online risk calculators outside of clinical consultations [1], it would be beneficial to improve the format of online health information so that unexpected or absent information is fully explained. This should include an explanation of why different risk calculators may produce different results, to increase awareness of accurate data entry for risk factors, and understanding of the assumptions behind the calculation [2]. Our findings suggest that people expect to be asked about broader aspects of their lifestyle and history than those included in the CVD risk models; consequently, the face validity is reduced by exclusion of these questions. The link between lifestyle advice and the risk factor questions also needs to be clear to avoid a negative reaction to the calculator. However, the aim of risk calculator tools will vary depending on the goals of the organization that develops them and the audience they are targeting, so our findings may have different implications for different designers.

### Strengths and Limitations

The strengths of this study include a novel topic and rigorous qualitative methods, including purposive sampling to achieve a heterogeneous patient sample, theme saturation, a trained interviewer, use of both semi-structured interview and think aloud data, and a framework analysis process that used multiple analysts and an iterative process to arrive at final themes. The external validity of the study is strengthened by the use of existing online risk calculators, self-reported risk factors, and widely used, validated Framingham risk equations. The limitations include missing video data for some participants and the possibility that the interview questions and presence of the interviewer may have influenced reactions to the websites. However, audio recordings were obtained for all participants, most users of such websites would have a prior interest in CVD, and the interviewer took care to avoid giving any interpretation of the results or reactions to the websites. The results may not reflect how consumers use risk calculators in a more realistic setting, and as typical with qualitative research, the sample was not designed to be representative of the general population but rather present a range of perspectives.

### Conclusions

Our findings demonstrate an interesting paradox: online heart age calculators are easily misunderstood and the results may be dismissed if the information is unexpected or negative, but the process of using such calculators may motivate lifestyle change regardless of the outcome. Future research should investigate both the benefits and harms of communicating risk in this way and how to increase the reliability and credibility of online health risk calculators.

## Multimedia Appendix 1

Protocol for data collection: think aloud method and semi-structured interview schedule.

## Multimedia Appendix 2

Structure of final analysis framework.

## Abbreviations

CVD: cardiovascular disease
GP: general practitioner
