# Supplementary material for: I Don't Believe It, But I'd Better Do Something About It: Patient Experiences of Online Heart Age Risk Calculators
Source: J Med Internet Res. 2014 May 5;16(5):e120. doi: 10.2196/jmir.3190 (PMC4026572; doi:10.2196/jmir.3190)
Supplement: Supplementary file 1 [file jmir_v16i5e120_app1.pdf]

THINK ALOUD INTERVIEW PROTOCOL

Suggest they might want to have a glass of water available while they do the interview, while you set up. Connect to the internet, open the three websites in separate windows, and set up a new recording in SMRecorder (set time to unlimited & specify file name).

Reiterate the patient information sheet:

Introduction: *Thank you for agreeing to be interviewed. As you know we are doing an interview study to explore patients' understanding of heart disease risk.*

Confidentiality/recording: *Everything you say will be strictly confidential. Your name will be removed from the written transcript, which will be made from an audio recording of the interview Any identifying names will be removed from the transcript. The interview includes looking at some online websites, and the computer screen will be video recorded so we can match the audio recording to what you were looking at. You will not be identifiable in the interview transcript or the video recording.*

The Interview: *The interview will last approximately 30-45 minutes. If you'd prefer not to answer any of the questions just let me know and we'll move on to the next question. You can also stop the interview at any time.*

Does that sound ok? Do you have any questions before we start?

Part 1. Understanding of heart disease risk

1. What do you think puts people at higher risk of developing heart disease?

2. Have you ever had any discussions with your doctor about heart disease risk or risk factors? If no - have you had any discussions about blood pressure or cholesterol? If yes – can you tell me about what your doctor said?

3. If doctor has discussed CVD risk with patient: How did your doctor explain heart disease risk to you? *[Prompt: did they use a graph or picture?]*

4. Would you say you are at low, medium or high risk of heart disease? Why?

5. How do you think your risk of heart disease compares to other people your age? Why?

Part 2. Heart age calculators

Introduction

In the next part of the interview, I will show you two online calculators that aim to help you understand your risk of heart disease.

Before we start, can you tell me how often you use the internet? *[If they use the internet a lot, give them more time to work out how to use the calculators themselves before giving guidance]*

What we'd like you to do is 'think aloud' about what you are doing as you go through the steps of each calculator, so we can understand the process you went through and what you thought about it. To help you get used to thinking aloud, we will do a practice exercise first, which is not related to heart disease. Then I will show you the two different calculators for heart disease risk. I won't be able to answer any questions about the actual task, just do whatever you think is best, and make sure you continue to think aloud the entire time. I will put a sign above the screen to remind you, and I will prompt you to keep talking if you are silent for more than 10 seconds. I will also ask you some questions at the end. Does that sound ok? Do you have any questions?

Practice Task (www.spotthedifference.com) ***** START RECORDER*****

Now we will do the practice task, which is a short 'spot the difference' game on the computer. It doesn't matter if you don't finish the game, I will tell you to stop once you've had enough practice thinking aloud. Start when you are ready, and remember to think aloud as you go.

[Prompt to keep talking, and give feedback on thinking aloud frequency at the end.]

Calculators (Unilever: www.heartagecalculator.com / NZ: www.knowyournumbers.co.nz)

Now I will show you the first/second calculator *[alternate order of calculators]*. Please follow the instructions on the screen and remember to think aloud as you go. I won't be able to answer any questions about the actual task, just do whatever you think is best. I will tell you when to stop, and then I will ask you some questions.

[Interviewer to note down heart age and percentage risk results, and whether smoking modification was used. Reset when they finish. Potential issues that may arise are listed below.]

Unilever: www.heartagecalculator.com.au

- loads slowly (open website before you start the interview)
- may come up with an 'insecure content' message (bring laptop + Telstra internet USB)
- need to provide metric height/weight/waist (bring conversion chart and tell them if needed)
- need to provide diastolic/stolic BP in right order (may need to tell them to reorder)
- continue button freezes in family history section (may need to start again or try different buttons)
- heart age result may not change if multiple factors adjusted (may need to reset heart age)
- if you press 'back' on browser you will need to restart (may need to tell them which button to use)

NZ: www.knowyournumbers.co.nz
- process is confusing (may need to tell them how to move on to the next step - click yellow arrow)
- results are confusing (may need to show them where the % and level of risk was explained during the final comparison questions, to get an idea of what format they prefer)
- can't go back to results after the risk factor modification page (stop them before they click arrow)

Questions after each calculator

1. What did you think of this calculator? [Prompt: what did you like or dislike about it?]
2. Can you explain the results of the calculator in your own words?
3. What does a heart age of x mean to you?
4. Why do you think your heart age is different from/the same as your current age?
5. Does a heart age of x mean that you are at low, medium or high risk of heart disease?
6. How do you think a heart age of x compares to other people your age?
7. The calculator allows you to change some factors to see how this affects your overall risk. What do you think will happen to your level of risk if you change your smoking status?
8. What do you think your heart age will be after you make this change?
9. Now change it on the screen to see what happens. Can you explain in your own words what happened when you changed your smoking status?

Part 3. Comparison of heart age calculators

Now to finish the interview, I will ask you a few questions about the two calculators you just used. I will just set them up so you can see them both, so you know which one I'm referring to.

1. Which calculator did you prefer, and why?

2. Why do you think the two calculators gave you a different heart age (if applicable)?

3. There were a few differences between the two calculators. I'm going to go through these and ask which one you preferred and why.

   [Note: use websites on computer screen to explain which calculator is being referred to]

   a. Calculator X showed your percentage level of risk as well as your heart age, but calculator Y only showed your heart age.
   - Which do you prefer, and why?
   - What does a percentage risk of x mean to you?

   b. Calculator X showed a graph, but calculator Y just gave written information.
   - Which do you prefer, and why?
   - What does the graph tell you?

   c. In calculator X, your heart age can only be the same as your current age or higher. In calculator Y, your heart age can be lower than your current age, if

your risk factors are very low.
- Which do you prefer, and why?

4. Did the calculators change what you thought about the causes of heart disease?

5. Did the calculators change what you thought about your own risk of heart disease?

6. Do you think these calculators are useful? [Prompt: who would they be useful for?]

7. How would you improve these calculators?

I would like to finish this interview by asking a few general questions about you. This will be used to describe the whole participant group, and will not be recorded with your interview [turn tape off].

1. What is your highest level of education?
- Year 10 or below
- Year 12
- Technical diploma
- Undergraduate university degree
- Postgraduate university degree

2. What is your marital status?
- Married
- De facto / living with partner
- Widowed
- Divorced
- Never married

3. In which country were you born? ___________________

4. If not Australia: In what year did you move to Australia to live? ___________________

5. Are you of Aboriginal and/or Torres Strait Islander origin? No Yes

6. Have you been diagnosed with any current medical conditions?
- No
- Yes (please specify):____

That's the end of the interview, thank you very much for your time. I will also give you an information sheet in case you would like more information about heart disease.
