# Supplementary material for: I Don't Believe It, But I'd Better Do Something About It: Patient Experiences of Online Heart Age Risk Calculators
Source: J Med Internet Res. 2014 May 5;16(5):e120. doi: 10.2196/jmir.3190 (PMC4026572; doi:10.2196/jmir.3190)
Supplement: Supplementary file 2 [file jmir_v16i5e120_app2.pdf]

Framework for analysis

Chart 1: Quantitative data

1. Main characteristics - gender, website order, heart age, absolute risk/level, heart age vs age
2. Unilever risk factor values - BP, chol, diabetes, smoking, fam hist, BMI, waist
3. New Zealand risk factor values - BP, chol, diabetes, smoking, fam hist, ethnicity
4. Data collection issues - internet use, think aloud quality, missing data

Chart 2: Initial risk perception

1. Perceived causes of CVD - risk factors
2. Perceived risk - risk level, what risk factors they attribute this to, what GP has said about this
3. Absolute risk of CVD - document if mentioned overall assessment or percentage
4. Risk level - low, mod, high and why
5. Risk comparison - risk compared to other people their age and why

Chart 3 & 4: Unilever & New Zealand websites

1. Think aloud - practical issues using calculator/website/computer (more superficial issues with usability)
2. Think aloud - emotional reaction to calculator/website (inc sponsor reaction, liking/not liking aspects)
3. Think aloud - cognitive thoughts about calculator/website (analysis of sponsor, info credibility, research)
4. Think aloud - emotional reaction to risk factors
5. Think aloud - cognitive thoughts about risk factors
6. Think aloud - emotional reaction to results
7. Think aloud - cognitive thoughts about/interpretation of results
8. Think aloud - emotional reaction to risk factor modification tool (own use of this)
9. Think aloud - cognitive thoughts about risk factor modification tool (own use of this)
10. Questions - impression of calculator
11. Questions - explanation of results
12. Questions - meaning of heart age
13. Questions - why heart age different from/same as current age
14. Questions - whether heart age indicates low/med/high risk
15. Questions - how heart age would compare to other people their age
16. Questions - reaction to/interpretation of smoking demonstration

Chart 5: Final impressions

1. Risk format feedback - thoughts about/preference for Unilever calculator
2. Risk format feedback - thoughts about/preference for New Zealand calculator
3. Risk format feedback - overall preference between calculators (if applicable)

4. Risk format feedback - reason for different calculator results (if applicable)
5. Risk format feedback - thoughts about/preference for heart age format
6. Risk format feedback - thoughts about/preference for NZ absolute risk percentage
7. Risk format feedback - thoughts about/preference for NZ graphical format
8. Risk format feedback - whether heart age should be less than real age if RFs are low
9. General feedback - whether calculators changed what they thought about the causes of CVD
10. General feedback - whether calculators changed what they thought about own CVD risk
11. General feedback - usefulness of calculators
12. General feedback - improvements to calculators
